# Carbon Dots Derived from the Maillard Reaction for pH Sensors and Cr (VI) Detection

**DOI:** 10.3390/nano10101924

**Published:** 2020-09-26

**Authors:** Zhi Ma, Yun Ma, Meiyu Gu, Xiyue Huo, Sainan Ma, Yini Lu, Yao Ning, Xuan Zhang, Bo Tian, Zhibiao Feng

**Affiliations:** 1 College of Food Science, Northeast Agricultural University, Harbin 150030, China; mxrex215@163.com (Z.M.); mayunneau@163.com (Y.M.); gumeiyu0913@163.com (M.G.); neauhuoxiyue@126.com (X.H.); neaumasainan@126.com (S.M.); lyn7310@163.com (Y.L.); nya163@163.com (Y.N.); zhang862129527@126.com (X.Z.); 2 Department of Chemistry, Northeast Agricultural University, Harbin 150030, China

**Keywords:** Maillard reaction, carbon dots, L-tryptophan, D-glucose, pH dependence, Cr(VI) detection

## Abstract

The Maillard reaction involves a series of complex reactions; fluorescent compounds have been considered as vital intermediate products of the reaction. In this article, carbon dots (CDs) based on the Maillard reaction (MR-CDs) were prepared with L-tryptophan and D-glucose, and they had excellent photoluminescence stability. MR-CDs showed stable pH-dependence behavior and exhibited an excellent linear response to pH in the range of 4.0–7.5 and 7.5–13.0, respectively. Under the masking effect of sodium fluoride for Fe(III), MR-CDs showed excellent selectivity and sensitivity for Cr (VI). The linear range of Cr(VI) was 0.2–50 μM and the limit of detection was 20 nM. (S/N ≥ 3). Furthermore, MR-CDs were used to detect Cr(VI) in tap water samples. The recoveries were between 95.8% and 98.94%, and RSDs were less than 3.17%.

## 1. Introduction

Carbon dots (CDs) are a class of carbon nanoparticles with particle sizes ranging from 1 to 10 nm. Due to their unique fluorescence properties, such as anti-photobleaching and high photoluminescence characteristics, they have been widely used as fluorescent probes [1,2,3]. Over the past years, different synthetic methods have been developed to prepare CDs. Generally, these methods are classified into hydrothermal synthesis, solvothermal synthesis, chemical oxidation and microwave/ultrasonic synthesis, etc. [4]. Although various materials have been used to prepare CDs, the synthesis strategy is still limited to screening small molecule organics for doping and little attention has been paid to endogenous fluorescence products from the Maillard reaction [5].

The Maillard reaction also called nonenzymatic browning, was first described by Louis-Camille Maillard in 1912 [6]. It is a series of parallel and continuous chemical reactions between the carbonyl-groups of reducing carbohydrates and the amino groups of amino compounds. It frequently occurs in the process of processing, cooking and storage of foods, in which a variety of reaction products are formed, including fluorescent compounds [7]. Fluorescence is characteristically produced during the intermediate stages of the Maillard reaction [8,9,10]. Recently, several reports showed that CDs could be prepared from the Maillard reaction, such as the pyrolysis process of chicken eggs, milk and orange juice [11,12,13]. Therefore, CDs can be prepared through controlling the process of the Maillard reaction. This reaction may provide a general method for the preparation of CDs.

In the past decade, emerging studies have shown that CDs used as fluorescence probes have drawn significant attention in the fields of metal ions detection and pH sensing due to their excellent optical properties. Chromium is a typical heavy metal pollutant in drinking water that is released through many industrial operations such as metallurgy, electroplating, pigments and leather tanning [14,15]. It exists in various oxidation states ranging from Cr(II) to Cr(VI). Among them, Cr(VI) is regarded as the most toxic, mutagenic and carcinogenic chemical state [16]. The permissible total chromium in drinking water recommended by the WHO is 50 μg/L (about 1 μM) [17]. Analytical methods such as atomic absorption spectroscopy, high-performance liquid chromatography and UV-visible spectroscopy, have been widely used to detect Cr(VI). Although these methods are sufficiently sensitive and selective, the complex pre-processing procedures, expensive equipment and time-consuming operation are still limitations to their applications. Fluorescence analysis has assumed an increasingly active role in quantitative analysis of trace metal ions due to its rapid response, high sensitivity and selectivity. For instance, Wang et al. synthesized nitrogen-doped CDs via hydrothermal treatment method to detect Cr(VI). The nitrogen-doped CDs can be selectively quenched by Cr(VI), and the detection limit was calculated as 4.16 μM [18]. Another related research was carried out by Zhang et al. [19]. The nitrogen and sulfur co-doped CDs were obtained by the pyrolysis process with citric acid and thiourea. A fluorescence assay was proposed for detection of Cr(VI) via the quenching of MR-CDs, and the limit of detection was 0.2 μM. Although various CDs were prepared to detect Cr(VI), it is still important to develop more sensitive CDs for determination of Cr(VI).

pH is a crucial target parameter in the industrial, biomedical and environmental applications [20]. Traditional small molecule pH probes usually suffer from relatively narrow and weak absorption spectra, toxicity concerns, poor emission performance and low photobleaching resistance [21]. The CDs as novel fluorescent nanomaterial has unique properties, especially low toxicity and biocompatibility. Furthermore, CDs are very sensitive to pH due to the abundant surface functional groups [22]. To date, quantities of CDs with neutral [23], acidic [24,25] or alkaline sensitivity [26] have been generally exploited to measure the pH of environmental or biologic samples. Nevertheless, most CDs suffer from the general problem of functioning over a small pH range so as to limit their applications. Therefore, it is essential to develop CDs as fluorescent pH probes to detect pH value with a wide range.

In this work, MR-CDs were prepared from the Maillard reaction model system of sugars and L-tryptophan. The factors, including type of sugars, molar ratio of L-tryptophan to D-glucose and pH value, were investigated. The particle size, optical characteristics, surface groups and elemental composition of MR-CDs were characterized, respectively. MR-CDs showed great pH-sensitive and reversible fluorescence in a wide pH range, which indicated that MR-CDs could be used as fluorescent pH probe for environmental samples and living cells. Moreover, we found that MR-CDs were highly selective to Cr(VI) and Fe(III). In the presence of sodium fluoride, the quenching effect of Fe(III) was masked, which allowed MR-CDs to be used as a sensitive fluorescent probe for the detection of Cr(VI) in aqueous media. This method was further used for detecting Cr(VI) in tap water samples.

## 2. Materials and Methods

### 2.1. Materials

L-tryptophan was obtained from Fine Chemical Research Institute (Tianjin, China). D-glucose and galactose were obtained from Bodi-chemical Technology Co., Ltd. (Tianjin, China). CuSO_4_, fructose and sucrose were purchased from Kermel Technology Co., Ltd. (Tianjin, China). K_2_Cr_2_O_7_, CrCl_3_, HgCl_2_, FeCl_3_, AlCl_3_, PbSO_4_, NaCl and KCl were bought from Benchmark Chemical Reagent Co., Ltd. (Tianjin, China). MgCl_2_ and BaCl_2_ were procured from Tianda Chemical Co., Ltd. (Tianjin, China). Ultrapure water was prepared through a Millipore Milli-Q gradient water purification system. All reagents were of analytical grade and were used directly without further purification.

### 2.2. Preparation of MR-CDs

L-tryptophan (0.1022 g, 0.5 mmol) and D-glucose (0.2702 g, 1.5 mmol) were dissolved in 30 mL ultrapure water. The pH value of the solution was tuned to 1.0 with HCl solution (37%) and then transferred the solution into a Teflon reactor. The reactor was heated from room temperature to 200 °C and held for 6 h. After cooling down to room temperature naturally, the product was centrifuged at 11,040 ×g for 30 min and the supernatant filtered with a microfiltration membrane. Then, the filtrate was dialyzed against ultrapure water using a dialysis bag (molecular weight cutoff, MWCO 3 kDa) for 48 h. MR-CDs powder was obtained via freeze-drying. Finally, dissolved 5 mg MR-CDs powder in 10 mL HCl–KCl solution (0.2 M, pH 2.5) to prepare MR-CDs solution (0.5 mg/mL) and stored at 4 °C for further use.

### 2.3. Characterization of MR-CDs

The surface morphologies of MR-CDs were characterized by a EM-2100F transmission electron microscope (JEOL Ltd., Tokyo, Japan). Absorption spectra were recorded on a UV-1660 UV-vis spectrophotometer (Shimadzu Corporation, Kyoto, Japan). Fluorescence excitation and emission spectra and intensity were recorded on a RF-6000 fluorescence spectrometer (Shimadzu Corporation, Kyoto, Japan). The Fourier-transform infrared (FTIR) spectra of MR-CDs were recorded on a Tensor-27 Fourier-transform infrared spectrometer (Bruker Corporation, Ettlingen, Germany). X-ray photoelectron spectroscopy (XPS) spectra were used to characterize the chemical composition using a K-Alpha X-ray photoelectron spectrometer (Thermo Fisher Scientific Inc., Waltham, MA, USA).

### 2.4. Quantum Yield Measurement of MR-CDs

The quantum yield (*QY*) of MR-CDs was determined using L-tyrosine as a reference (dissolved in water, *QY* = 14%, excited at 300 nm) [20]. Equation (1) was used to determine the quantum yield:

(1) $$QY_{S}={QY}_{R}\;\times \;\frac{F_{S}}{F_{R}}\;\times \;\frac{A_{R}}{A_{S}}$$

where *S* and *R* represent the substance and the reference (L-tyrosine), *F* represents the integrated fluorescence area and A represents the absorption value measured at 300 nm.

### 2.5. pH Response of MR-CDs

The solutions with different pH values were prepared with HCl-KCl, citric acid-Na_2_HPO_4_, Na_2_B_4_O_7_ and NaHCO_3_-NaOH, then 100 μL stock solution of MR-CDs was added into 10 mL solutions of different pH value (pH 1.0–13.0). Then, 10 min later, their fluorescence emission spectra were recorded under 300 nm excitation.

### 2.6. Detection of Cr (VI)

Cr(VI) solutions of different concentrations were prepared with HCl-KCl solution (0.2 M, pH 2.5). Then 100 μL MR-CDs stock solution was added to the Cr(VI) solutions. After 10 min, their fluorescent emission spectra were measured at the excitation wavelength of 300 nm. All analyses were conducted in triplicate.

The calibration curve was obtained by the Stern–Volmer formula:

(2) $$\frac{F_{0}}{F}=K_{\mathrm{sv}}\left[\mathrm{Cr}(\mathrm{VI})\right]+1$$

where *K*_SV_ is the quenching constant, *F*_0_ and *F* represent the fluorescence intensity of MR-CDs absence and presence of Cr (VI).

### 2.7. Samples Pretreatment

Tap water was obtained from the laboratory of Northeast Agricultural University (China). In brief, 1 mL sodium fluoride (1 mM) was added into 9 mL tap water and mixed well by magnetic stirring. Then, the solution was centrifuged at 11,040× *g* for 10 min and the supernatant was filtered with a 0.45 µm membrane.

## 3. Results

### 3.1. Preparation of MR-CDs

**Figure 1:**
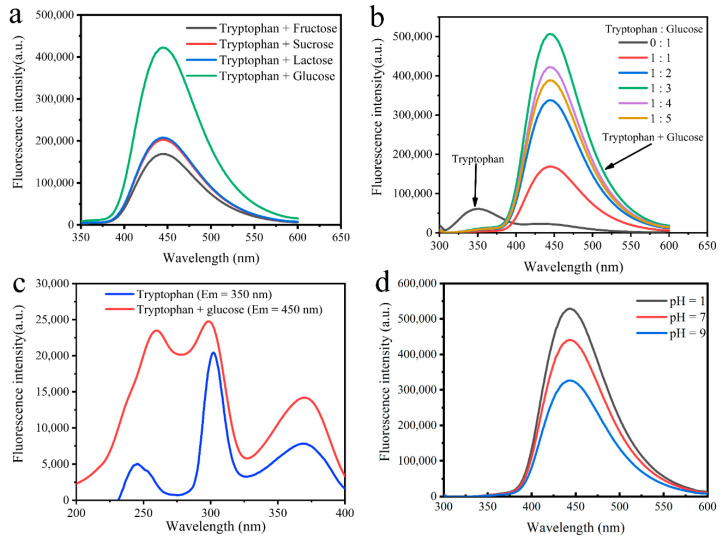
(**a**) Fluorescence emission spectra of carbon dots (CDs) based on the Maillard reaction (MR-CDs) prepared with L-tryptophan and different sugars; (**b**) fluorescence emission spectra of MR-CDs prepared from L-tryptophan and D-glucose with various molar ratios; (**c**) fluorescence excitation spectra of MR-CDs with or without D-glucose (emission wavelength was 450 nm and 350 nm); (**d**) fluorescence emission spectra of MR-CDs prepared at various pH values.

The fluorescence intensities of MR-CDs were influenced by the type of sugars (D-fructose, sucrose, D-galactose and D-glucose), molar ratio of L-tryptophan to sugars and pH of reaction environment. As shown in Figure 1a, higher fluorescence intensity was observed in the L-tryptophan-D-glucose Maillard reaction system than in other L-tryptophan-sugars reaction system. Figure 1b showed the effect of molar ratio of L-tryptophan to D-glucose on the fluorescence intensity of MR-CDs. The addition of D-glucose increased the fluorescence intensity until the molar ratio of L-tryptophan to D-glucose reached 1:3, however, more addition of D-glucose showed an opposite effect on the fluorescence intensity. Furthermore, the emission maxima of MR-CDs red-shifted from 350 to 450 nm when sugars were incorporated into L-tryptophan. In general, the emission maximum of MR-CDs is related to the energy spacing between the ground state and excited state vibrational energy levels, and the energy spacing is dependent upon the chemical structure of fluorophore [27]. The excitation spectra of MR-CDs are shown in Figure 1c. MR-CDs with and without D-glucose incorporated emitted at 450 nm and 350 nm, respectively. They had different absorption bands of π–π* transition at 245 nm. For MR-CDs introduced D-glucose, the absorption band at 245 nm showed relatively high absorbance and red-shift, which indicated that the introduced of D-glucose extended the conjugated π– electron system of MR-CDs. The conjugated π electron system intensely coupled with surface electronic states and further reduced the energy gap for the π–π* transitions. The lower energy gap enhanced the fluorescence intensity and induced a red-shift [3].

Figure 1d shows the fluorescence emission spectra of MR-CDs prepared in various pH environments. The fluorescence intensity of MR-CDs decreased while the pH changed from 9.0 to 1.0, which indicated that the form of MR-CDs strongly depended on the pH value. In general, the fluorescent compounds formed in the Maillard reaction can be regarded as intermediates during the formation of melanoidins and are also considered as the indicators to detect the early stage of the Maillard reaction [9,28,29]. As shown in the general scheme of the Maillard reaction [30] (Figure S1), the initial Maillard reaction comprises the carbonyl-amino condensation and the formation of Amadori products [31,32]. The process of Amadori rearrangement involves protonation of the nitrogen atoms of glucosyl-amine, and therefore, low pH may induce the formation of fluorescence products [33]. The advanced and final Maillard reaction involves two processes, one is the degradation of Amadori products into sugar–amino compounds, the other is the condensation between amino compounds and sugar fragments into brown pigments (melanoidins) [34]. The brown pigments and fluorescence products are two main components of the advanced Maillard reaction. In this stage, the initial products of condensation are fluorescent, and continuation of the reaction leads to the formation of melanoidins [31,33]. In this work, the lower the pH was, the more protonated amino groups were present in the Maillard reaction system, and thus, the amino groups were less reactive with sugars. As a result, the low pH inhibited the formation of melanoidins, and the fluorescent products were also accumulated [34,35].

### 3.2. Characterization of MR-CDs

**Figure 2:**
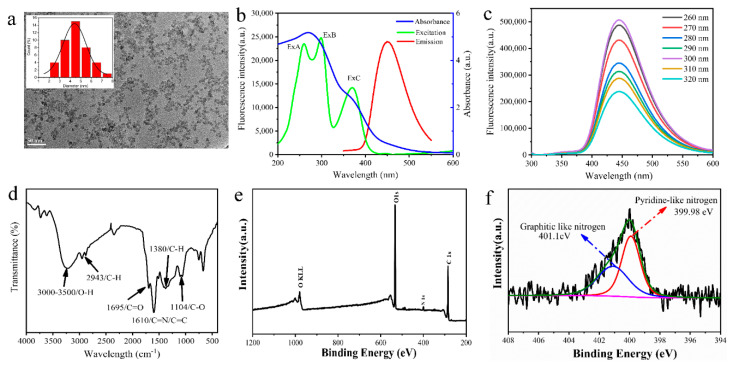
(**a**) TEM image (inset: particle size distribution); (**b**) UV-vis absorption spectrum, fluorescence excitation spectrum (λ_em_ = 450 nm) and emission spectrum (λ_ex_ = 300 nm); (**c**) fluorescence emission spectra at different excitation wavelengths; (**d**) FTIR spectrum; (**e**) XPS spectrum; (**f**) high-resolution XPS spectrum of N1s.

As shown in Figure 2a, MR-CDs displayed a near sphere structure and the average diameter was about 4–5 nm.

The optical properties of MR-CDs were explored through UV-vis absorption and fluorescence excitation and emission spectroscopy. As shown in Figure 2b, the UV-vis absorption spectrum showed two absorption bands at 270 nm and 350 nm, resulting from the π–π* electronic transition of C=C and the n–π* transition of surface groups (C=O and C=N, etc.), respectively [36,37]. The fluorescence emission spectrum showed three absorption peaks at 258 nm (Ex A), 300 nm (Ex B) and 370 nm (Ex C), which were consistent with the results of the UV-visible absorption spectrum. The MR-CDs obtained the largest Stokes shift of 150 nm at excitation wavelength of 300 nm, which minimized the self-absorption and reduced the excitation interference. As shown in Figure 2c, the peak position of fluorescence emission spectrum of MR-CDs remained almost unchanged when the excitation wavelength changed from 260 to 320 nm, which suggested that the MR-CDs showed excitation wavelength-independent photoluminescence behavior. This behavior was also reflected in the three-dimensional spectrum (Figure S2), which exhibited three emission peaks, one characteristic peak at 450 nm and two weak scattering peaks over 300–400 nm. In addition, the QY of MR-CDs was calculated to be 18%, which was comparable to those of most amino acid-derived CDs reported previously [38,39,40].

The surface functional groups of MR-CDs were investigated using Fourier-transform infrared spectroscopy measurement. As can be seen in Figure 2d, the broad absorption peak from 3000–3500 cm^−1^ was ascribed to O–H stretching vibration, which indicated there were many associative hydroxyl groups existing on the surface of MR-CDs [37]. The sharp peak at 1610 cm^−1^ was attributed to the stretching vibration of C=C/C=N bonds [41]. The medium intensity absorption peaks centered at 1380 cm^−1^ and 2943 cm^−1^ were ascribed to the C–H bending vibration. The absorption peaks at 1104 cm^−1^ and 1695 cm^−1^ were ascribed to the C–O and C=O stretching vibration, respectively [42].

The elements and functional groups information of MR-CDs were further confirmed by XPS analysis. As exhibited in Figure 2e, the full XPS spectrum of MR-CDs showed three typical binding energy peaks at 285.08 eV, 400.08 eV and 533.08 eV, indicating that MR-CDs mainly consisted of C (64%), O (33%), N (3%). The C1s high-resolution spectrum (Figure S3) revealed three blinding peaks at 284.73 eV, 285.78 eV and 288.33 eV, which were attributed to C–C, C–O/C–N and C=O bands, respectively. The high-resolution spectrum of O1s (Figure S3) showed one peak at 532.63 eV, which was assigned to C=O bands. Moreover, the N1s spectrum (Figure 2f) could be deconvoluted into two peaks at 399.98 eV and 401.13 eV, which were pyrrolic-like N and graphitic N, respectively [43]. The presence of pyrrolic-like N could contribute to the π–conjugated system with a pair of π-electrons in MR-CDs [44]. The analyses of FTIR and XPS suggested that the surface of MR-CDs had sufficient oxygen-containing functional groups, such as–OH and–COOH groups, and these groups gave the MR-CDs excellent water solubility.

### 3.3. Stability of MR-CDs

**Figure 3:**
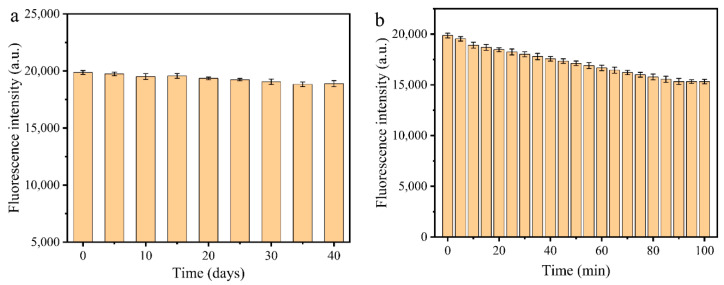
Fluorescence stability of MR-CDs. (**a**) Storage time in dark condition; (**b**) UV-illumination time.

Fluorescence stability of MR-CDs is important for their applications. To evaluate the photostability of MR-CDs, they were stored at room temperature in the dark, and the fluorescence intensities of MR-CDs were investigated every five days. As shown in Figure 3a, there was hardly any variation in fluorescence intensity, which enables the MR-CDs as fluorescent probe for long-term measurement. In addition, the photobleaching resistance of MR-CDs was measured through continuously irradiating with 300 nm ultraviolet light, and the fluorescence intensity of MR-CDs was recorded every five minutes. As shown in Figure 3b, the fluorescence intensity of MR-CDs changed slightly and kept about 80% of the initial intensity even after 100 min irradiation. The photobleaching resistance was higher than that of the conventional fluorophores, indicating the MR-CDs can be used as an alternative for fluorescence detection [45,46].

### 3.4. pH-Dependence of MR-CDs

**Figure 4:**
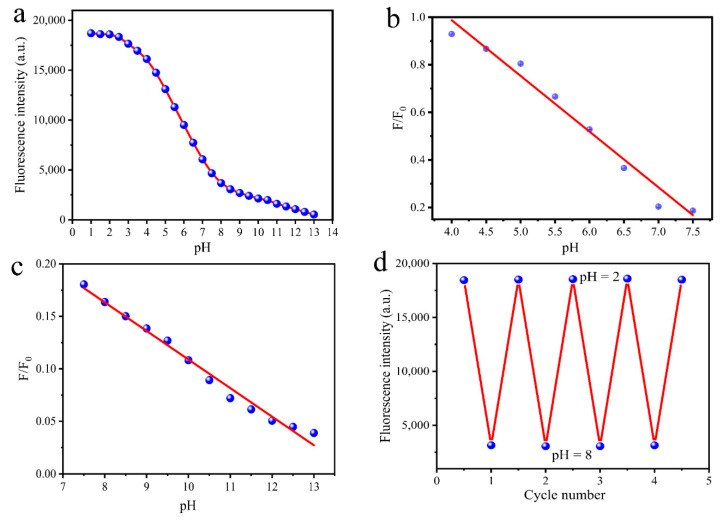
(**a**) Fluorescence intensity of MR-CDs with different pH values. Calibration curve of quenching efficiency (*F*/*F*_0_) and pH with a range from (**b**) 4.0–7.5 and (**c**) 7.5–13.0; (**d**) fluorescence reversibility of MR-CDs.

In order to research the fluorescence responses of MR-CDs to pH, the fluorescence pH titration experiment of MR-CDs was investigated. From Figure 4a, MR-CDs showed the strongest fluorescence when pH value ranged from 1.0 to 3.0. However, fluorescence intensity decreased dramatically when pH value raised from 3.0 to 13.0 (approximately 19-fold decrescent for MR-CDs). This indicated that MR-CDs were extremely sensitive to protons. Figure 4b,c displays the plots of their fluorescence-quenching efficiency versus pH value in the range of 4.0–7.5 and 7.5–13.0 (Table 1). The fluorescence reversibility is essential for MR-CDs as a reliable pH probe. The pH response reversibility was tested when pH was cycled between 2.0 and 8.0. As shown in Figure 4d, MR-CDs showed excellent fluorescence reversibility and photostability, which is more favorable for real-time monitoring of pH fluctuation.

The relationship between pH value and fluorescence intensity at 450 nm can be expressed by the Henderson–Hasselbalch-type mass action equation [47],

(3) $$\log\left[\frac{F_{\max}-F}{F-F_{\min}}\right]=\mathrm{pH}-pKa$$

where *F* is the observed fluorescence intensity of MR-CDs; *F*_max_ and *F*_min_ are the fluorescence intensity of MR-CDs in its acid (pH 3.5) and conjugate base (pH 7.5), respectively. The p*K*a value of MR-CDs was calculated to be 5.75 ± 0.03 (Figure S4), which indicated that MR-CDs are suitable for studying acidic organelles.

The XPS spectra of MR-CDs showed that there were two different chemical states of nitrogen on the surface of MR-CDs, pyridinic nitrogen and graphitic nitrogen. However, only pyridine nitrogen groups (C=N–C) can accept protons [48]. The variation of fluorescence versus pH could be ascribed to the reversible protonation and deprotonation of the pyridine nitrogen. The decrease of pH-induced protonation of pyridine nitrogen atoms of MR-CDs. Subsequently, the protons transferred from protonated nitrogen atoms to conjugated carbon structure, which increased the fluorescence intensity of MR-CDs [49]. To reveal the pH-response mechanism of CDs, zeta potentials of CDs with different pH values were investigated. As shown in Figure S5, with the increase of pH value, zeta potentials of CDs decreased form +5.4 mV to −29.9 mV. The isoelectric point of the CDs was determined to be 4.5. The zeta potentials of CDs in strong alkaline conditions showed pH-induced deprotonation of pyridine nitrogen groups, which can oppose aggregation by electrostatic repulsion [50].

In addition, in acidic medium, the formation of hydrogen bonds of carboxyl and hydroxyl groups increased the conformational rigidity of MR-CDs and therefore promoted fluorescence emission as well [51]. The fluorescence-quenching of MR-CDs at high pH value, may be attributed to the deprotonation of pyridinic nitrogen atoms, which enlarged the band gap energy of MR-CDs and caused fluorescence to be weak under alkaline conditions [52].

### 3.5. Selectivity of MR-CDs

**Figure 5:**
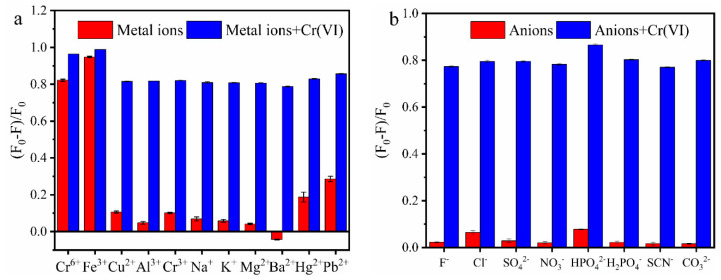
Fluorescence-quenching efficiency and interference effects of various metal and anion ions, where *F*_0_ and *F* are the fluorescence intensity of MR-CDs in the nonexistence and existence of various interfering ions under the excitation wavelength of 300 nm. (**a**) Metal ions; (**b**) anions.

To evaluate the selectivity of MR-CDs, different kinds of metal ions and anions with same concentration (100 μM) were added to the MR-CDs stock solution, then the fluorescence-quenching efficiency of MR-CDs was measured. From Figure 5a,b, compared with other ions, Cr(VI) and Fe(III) showed significantly quenching effect on MR-CDs. Except for Fe(III) ions, other co-existence ions exhibited negligible influence on the fluorescence-quenching of Cr(VI) to MR-CDs. The interference of Fe (III) on the determination of Cr(VI) could be eliminated through adding sodium fluoride solution to the samples. As exhibited in Figure S6 with the addition of sodium fluoride, the fluorescence quenched by Fe(III) was restored, while the fluorescence quenched by Cr(VI) was not restored. Furthermore, when sodium fluoride was added to the MR-CDs solution containing Fe(III) and Cr(VI), their fluorescence intensity showed little difference from the MR-CDs solution only containing Cr(VI).

### 3.6. Detection of Cr (VI)

**Figure 6:**
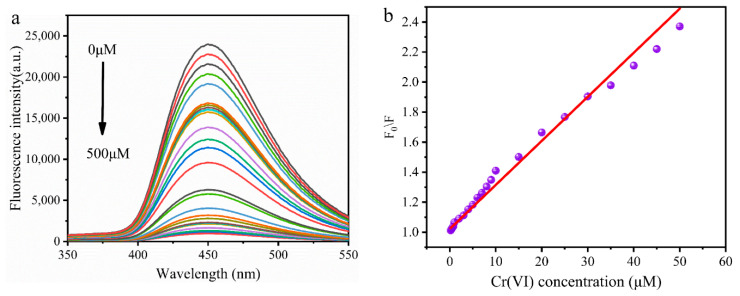
(**a**) Emission spectra of MR-CDs solution with various concentrations of Cr(VI) (from top to bottom: 0, 0.2, 0.4, 0.6, 0.8, 1.0, 2.0, 4.0, 6.0, 8.0, 10, 20, 30, 40, 50, 100, 200, 300, 400 and 500 μM); (**b**) plot of the quenching efficiency against the concentration of Cr (VI).

The MR-CDs were used to detect Cr(VI) in aqueous solution. Because MR-CDs exhibited stronger fluorescence intensity in acidic conditions, Cr(VI) ions were determined at pH 2.5. As shown in Figure 6a, the fluorescence intensity of MR-CDs at 450 nm reduced with the concentrations of Cr(VI) from 0 to 500 μM. The curve of fluorescence-quenching efficiency versus the concentration of Cr(VI) was plotted in Figure 6b. The fluorescence-quenching efficiency of MR-CDs showed a linear relationship with the concentration of Cr(VI) within the range of 0.2 to 50 μM. The calibration equation *F*_0_/*F* = 0.03 [Cr(VI)] + 1.02 (R^2^ = 0.988) was developed and the limit of detection (LOD) was 20 nM (S/N ≥ 3). As listed in Table 2, the LOD was lower than that reported previously, and also lower than the standard for drinking water quality of the WHO.

### 3.7. Practical Application

Cr(VI) in water supply systems is almost all from fixtures, faucets and pipes that contain chromium. In this study, MR-CDs were applied to detect Cr(VI) in tap water samples. After adding 1.0, 5.0 and 10.0 μM Cr(VI) into tap water, recovery assays were performed. As can be seen in Table 3, the recoveries were between 95.8% and 98.94%, and relative standard deviations (RSDs) were less than 3.17%. The results indicated that the MR-CDs are suitable for quantitative determination of Cr(VI) in water samples.

## 4. Conclusions

MR-CDs were prepared through hydrothermal treatment of L-tryptophan and D-glucose at pH 1.0. Compared with L-tryptophan, the introduction of D-glucose significantly improved the fluorescence of MR-CDs. Furthermore, low pH promoted the formation of fluorescent products and inhibited their consumption by influencing certain stages of the Maillard reaction. The MR-CDs showed excellent photostability with the quantum yield of 18%. Reversible protonation and deprotonation of pyridine nitrogen resulted in the fluorescence intensity of MR-CDs sensitive to pH variation. The fluorescence-quenching efficiency of MR-CDs showed a broad linear relationship with the pH value in the range of 4.0 to 7.5 and 7.5–13.0, respectively. Based on the masking of sodium fluoride on Fe(III), the MR-CDs exhibited great selectivity to Cr(VI). On this basis, MR-CDs showed a wide linear range for Cr(VI) within the concentration of 0.2–50 μM and had a low detection limit of 20 nM. Furthermore, the MR-CDs were used to detect Cr(VI) in tap water samples. The recoveries were between 95.8% and 98.94%, and RSDs were less than 3.17%.

## Figures and Tables

**Table 1:** Regression equation between quenching efficiency and pH.

pH Linear Range	Regression Equation	Correlation Coefficient (R^2^)
4.0–7.5	*F*/*F*_0_ = −0.2342 pH + 1.9245	0.984
7.5–13.0	*F*/*F*_0_ = −0.0272 pH + 0.3182	0.985

**Table 2:** Comparison of different CDs for the detection of Cr(VI).

Methods	Materials	Linear Range (μM)	LOD(μM)	Citations
N, S-CDs	Citric acid and thiourea	1–10	0.20	[19]
N-CDs	Citric acid and glycine	5–200	4.16	[18]
N, S-CDs	Thiourea and citric acid	1–10	0.2	[19]
Green synthesis-CDs	Peanut	2–10	1.9	[53]
GQDs	g-C3N4	0.6–300	0.15	[54]
MR-CDs	D-glucose and L-tryptophan	0.2–50	0.02	This work

**Table 3:** Recoveries of Cr(VI) in tap water samples detected by the proposed method (*n* = 5).

Samples	Added (uM)	Found (uM)	Recovery (%)	RSD (%)
1	0	Not detected	–	–
2	1.0	0.958	95.8	3.17
3	5.0	4.809	96.18	2.63
4	10.0	9.894	98.94	1.70

## Supplementary Materials

The following are available online at https://www.mdpi.com/2079-4991/10/10/1924/s1, Figure S1: General scheme of the Maillard reaction adapted from Hodge. Figure S2: Three-dimensional spectrum of MR-CDs. Figure S3. (a) High-resolution spectrum of C1s; (b) high-resolution spectrum of O1s. Figure S4: Fitting curve of the Henderson–Hasselbalch equation (*R*^2^ = 0.99). Figure S5: Zeta potentials of MR-CDs in solutions of different pH value. Figure S6. (a) Fluorescence emission spectra of MR-CDs with Fe^3+^, Cr^6+^ and F^-^ ions (the concentration of Fe^3+^, Cr^6+^ and F^-^ were 100 μM, 100 μM and 1 mM, respectively); (b) fluorescence-quenching efficiency of various ions. (0: MR-CDs, 1: MR-CDs+Fe^3+^, 2: MR-CDs+Cr^6+^, 3: MR-CDs+Fe^3+^+Cr^6+^, 4: MR-CDs+Fe^3+^+F^-^, 5: MR-CDs+Fe^3+^+Cr^6+^+F^-^).
