# Supplementary material for: Carbon Dots Derived from the Maillard Reaction for pH Sensors and Cr (VI) Detection
Source: Nanomaterials (Basel). 2020 Sep 26;10(10):1924. doi: 10.3390/nano10101924 (PMC7599493; doi:10.3390/nano10101924)
Supplement: Supplementary file 1 [file nanomaterials-10-01924-s001.pdf]

nanomaterials

Supplementary materials

Carbon dots derived from Maillard reaction for pH sensors and Cr (VI) Detection

Zhi Ma [1], Yun Ma [1], Meiyu Gu [1], Xiyue Huo [1], Sainan Ma[1], Yini Lu [1], Yao Ning [1], Xuan Zhang [1], Bo Tian [1,*] and Zhibiao Feng [2,*]

[1] College of Food Science, Northeast Agricultural University, Harbin,150030, China; mazhi215@outlook.com (Z.M.); 1534320711@qq.com (Y.M.); gumeiyu0913@163.com (M.G.); neauhuoxiyue@126.com (X.H.); neaumasainan@126.com (S.M.); 947946184@qq.com (Y.L.); nya163@163.com (Y.N.); zhang862129527@126.com (X.Z.); tianbo@neau.edu.cn (B.T.);

[2] Department of Chemistry, Northeast Agricultural University, Harbin,150030, China; fengzhibiao@neau.edu.cn (Z.F.)

* Correspondence: tianbo@neau.edu.cn (B.T.); fengzhibiao@neau.edu.cn (Z.F.)Tel.: +86-451-5519-04-79 (B.T.); Tel.: +86-451-5519-02-22 (Z.F.)

Figure S1: The general scheme of the Maillard reaction adapted from Hodge[1].

Figure S2: The three-dimensional spectrum of MR-CDs.

Figure S3: (**a**) The high-resolution spectrum of C1s. (**b**) The high-resolution spectrum of O1s.

Figure S4: Fitting curve of the Henderson-Hasselbalch equation (R2 = 0.99).

Figure S5: Zeta potentials of MR-CDs in buffer solutions of different pH value.

Figure S6: (**a**) Fluorescence emission spectra of MR-CDs with Fe3^{+}, Cr6^{+} and F^{-} ions (the concentration of Fe^{3+}, Cr^{6+} and F^{-} were 100 μM, 100 μM and 1 μM, respectively). (**b**) Fluorescence quenching efficiency of various ions. (0: MR-CDs, 1: MR-CDs+Fe^{3+}, 2: MR-CDs+Cr^{6+}, 3: MR-CDs+Fe^{3+}+Cr^{6+}, 4: MR-CDs+Fe^{3+}+F^{-}, 5: MR-CDs+Fe^{3+}+Cr^{6+}+F^{-})

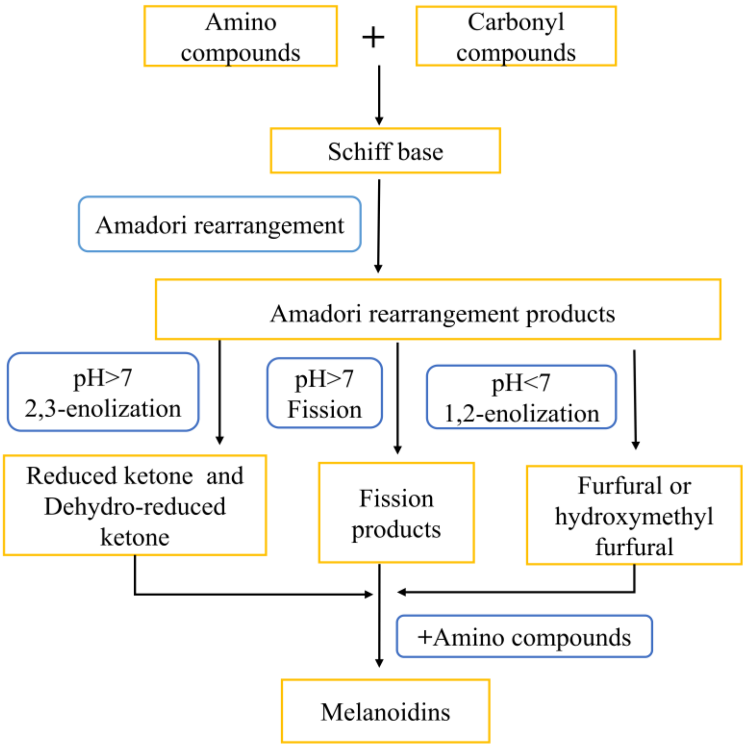

Figure S1. The general scheme of the Maillard reaction adapted from Hodge[1].

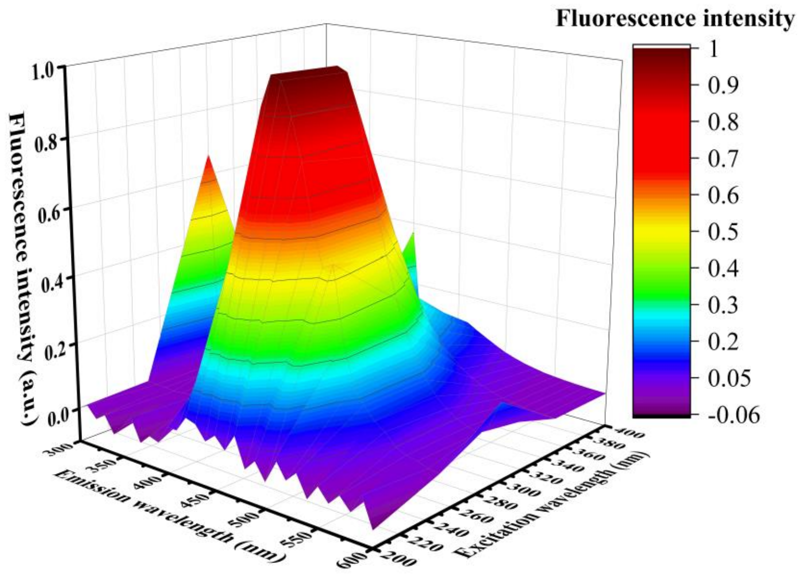

Figure S2. The three-dimensional spectrum of MR-CDs.

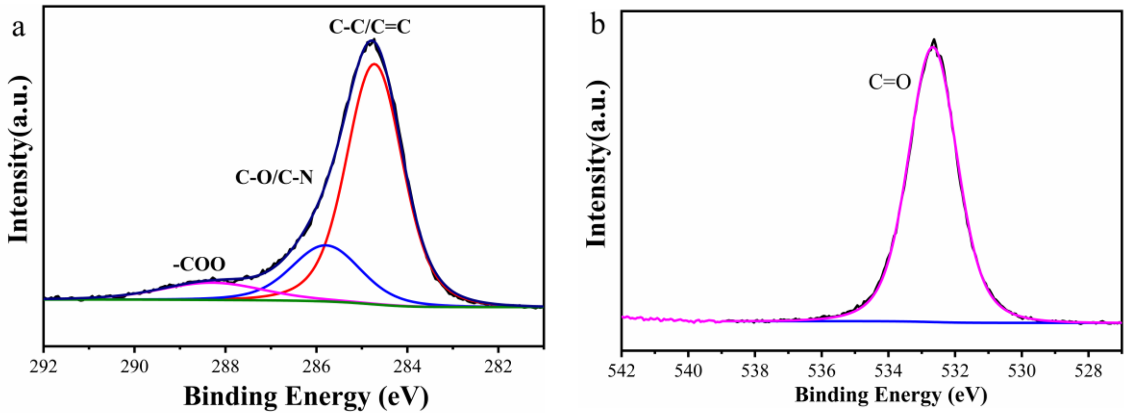

Figure S3. (**a**) High-resolution spectrum of C1s. (**b**) High-resolution spectrum of O1s.

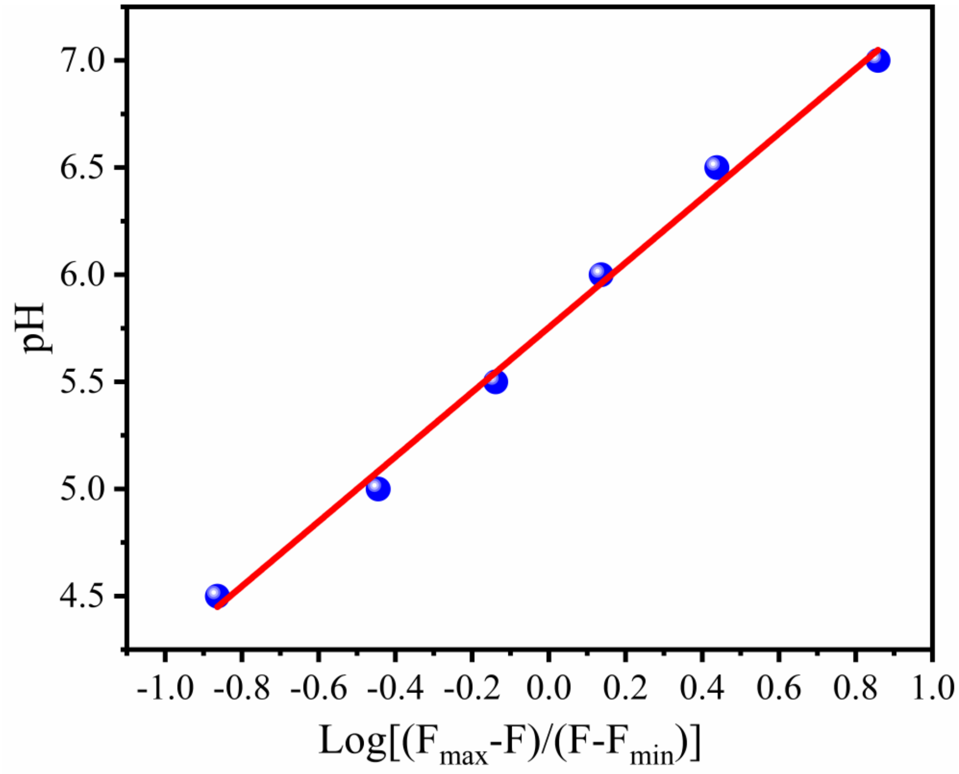

Figure S4. Fitting curve of the Henderson-Hasselbalch equation (R^2 = 0.99).

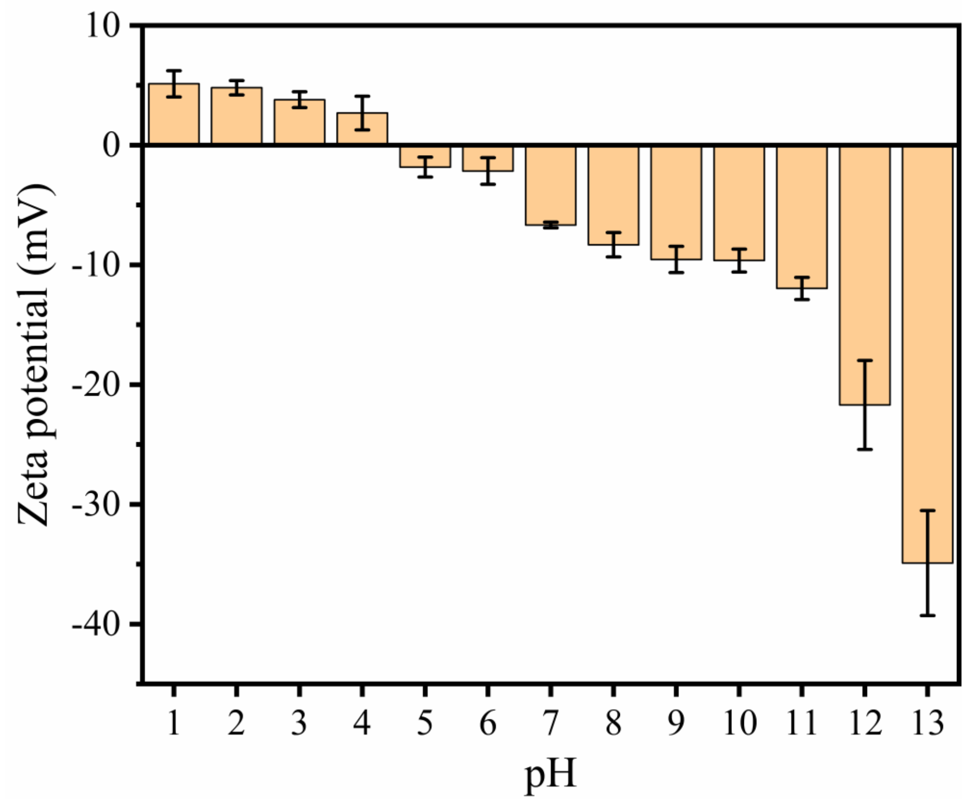

Figure S5. Zeta potentials of MR-CDs in buffer solutions of different pH value.

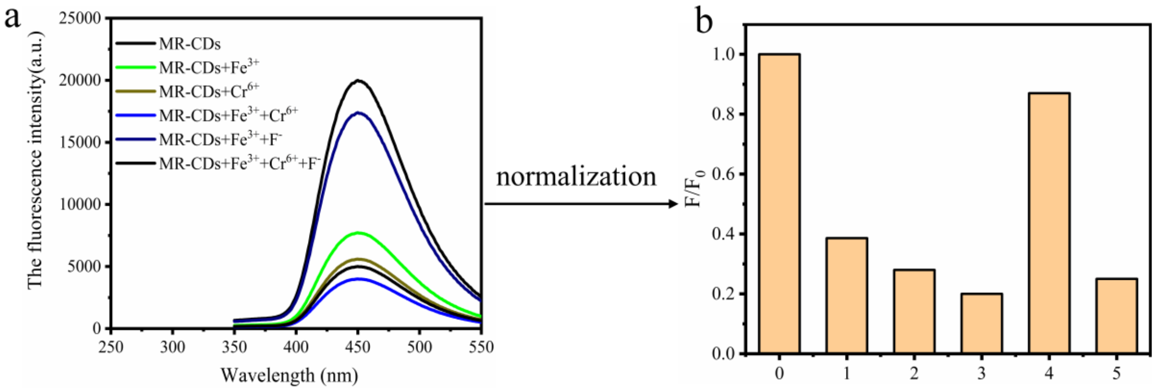

Figure S6. (**a**) Fluorescence emission spectra of MR-CDs with Fe^{3+}, Cr^{6+} and F^- ions (the concentration of Fe^{3+}, Cr^{6+} and F^- were 100 μM, 100 μM and 1 μM, respectively). (**b**) Fluorescence quenching efficiency of various ions. (0: MR-CDs, 1: MR-CDs+Fe^{3+}, 2: MR-CDs+Cr^{6+}, 3: MR-CDs+Fe^{3+}+Cr^{6+}, 4: MR-CDs+Fe^{3+}+F^-, 5: MR-CDs+Fe^{3+}+Cr^{6+}+F^-)

References

1. Hodge, J.E. Dehydrated Foods, Chemistry of Browning Reactions in Model Systems. *Journal of Agricultural and Food Chemistry* **1953**, *1*, 928-943, doi:10.1021/jf60015a004.
